# The Role of *Legionella pneumophila* Serogroup 1 Lipopolysaccharide in Host-Pathogen Interaction

**DOI:** 10.3389/fmicb.2019.02890

**Published:** 2019-12-17

**Authors:** Marta Palusinska-Szysz, Rafal Luchowski, Wieslaw I. Gruszecki, Adam Choma, Agnieszka Szuster-Ciesielska, Christian Lück, Markus Petzold, Anna Sroka-Bartnicka, Bozena Kowalczyk

**Affiliations:** ^1^Department of Genetics and Microbiology, Faculty of Biology and Biotechnology, Institute of Biological Sciences, Maria Curie-Skłodowska University, Lublin, Poland; ^2^Department of Biophysics, Faculty of Mathematics, Physics and Computer Science, Institute of Physics, Maria Curie-Skłodowska University, Lublin, Poland; ^3^Department of Virology and Immunology, Faculty of Biology and Biotechnology, Institute of Biological Sciences, Maria Curie-Skłodowska University, Lublin, Poland; ^4^National Reference Laboratory for Legionella, Institute of Medical Microbiology and Hygiene, University of Technology Dresden, Dresden, Germany; ^5^Department of Infectious Diseases, Medical Microbiology and Hygiene, Heidelberg University Hospital, Heidelberg, Germany; ^6^Department of Biopharmacy, Medical University of Lublin, Lublin, Poland

**Keywords:** *Legionella pneumophila*, lipopolysaccharide, adhesion, atomic force microscopy, FLIM microscopy

## Abstract

The *Legionella pneumophila* TF3/1 mutant of the Corby strain, which possesses a point mutation in the active site of the *O*-acetyltransferase, synthesized the polysaccharide chain with a reduced degree of substitution with *O*-acetyl groups. The mutant did not produce a high-molecular-weight lipopolysaccharide (LPS) fraction above 12 kDa. The disturbances in LPS synthesis have an effect on the composition of other macromolecules (lipids and proteins), as indicated by differences in the infrared absorption spectra between the *L. pneumophila* Corby strain and its TF3/1 mutant. The wild-type strain contained less N^+^–CH_3_ and C-N groups as well as more CH_3_ groups than the mutant. The fatty acid composition showed that the wild type strain synthesized more branched acyl residues (*a*15:0, *i*16:0, and *a*17:0), a less unsaturated acid (16:1), and a straight-chain acid (18:0) than the mutant. The mutant synthesized approximately twice more a long-chain fatty acid (20:0) than the wild type. The main differences in the phospholipids between both strains were found in the classes of phosphatidylcholines and phosphatidylglycerols (PG). Substantial differences in the cell surface topography of these bacteria and their nanomechanical properties were shown by atomic force microscopy (AFM). The wild type strain had no undulated surface and produced numerous vesicles. In the case of the mutant type, the vesicles were not numerous, but there were grooves on the cell surface. The average roughness of the cell surface of the mutant was approximately twofold higher than in the wild-type strain. In turn, the wild-type strain exhibited much better adhesive properties than the mutant. The kinetic study of the interaction between the *L. pneumophila* strains and *Acanthamoeba castellanii* monitored by Förster resonance energy transfer revealed a pronounced difference, i.e., almost instantaneous and highly efficient binding of the *L. pneumophila* Corby strain to the amoeba surface, followed by penetration into the amoeba cells. This process was clearly not as efficient in the case of the mutant. The results point to LPS and, in particular, to the length of the polysaccharide fraction as an important *L. pneumophila* determinant involved in the process of adhesion to the host cell.

## Introduction

*Legionella pneumophila* is an intracellular pathogen and the main causative agent of Legionnaires’ disease – a severe and often fatal pneumonia. Although one case of human-to-human transmission of *L. pneumophila* has been reported (Correia et al., 2016), the vast majority of evidence indicates that human infection (sporadic cases or epidemic outbreaks) is most frequently caused through inhalation of bacteria-contaminated water distributed as a water-air aerosol by air-conditioning systems, cooling towers, industrial and medical facilities, and sanitary network devices (van Heijnsbergen et al., 2015). The bacteria infect both mammalian cells (alveolar macrophages) and environmental hosts, such as amoeba. Inside host cells, the bacteria withstand the multifaceted defenses of the phagocyte and replicate within a unique membrane-bound compartment, i.e., the *Legionella*-containing vacuole (LCV). Formation of the replicative vacuole mainly requires an efficient Dot/Icm type IV secretion system, which has been relatively well characterized (Rolando and Buchrieser, 2014). Besides proteins, bacterial surface structures are involved in highly specific interactions, which can determine *Legionella* survival in contact with the host cell. However, the exact role of these structures in bacterial cell adaptation is poorly understood. *L. pneumophila* do not express a capsule or an exopolysaccharide. Therefore, the lipopolysaccharide (LPS) localized in the outer membrane is the predominant molecule on the cell surface of these bacteria that contributes to the cell surface properties in an exceptionally important way.

The chemical structure of *L. pneumophila* LPS is different from that of the endotoxins of other Gram-negative bacteria, despite the similar structure. This multi-functional macromolecule is composed of a polysaccharide part: an O-specific chain, an outer and inner core, and a lipid part, i.e., lipid A. The saccharide backbone of *L. pneumophila* lipid A is composed of 2,3-diamino-2,3-dideoxy-D-glucose disaccharide linked via an amide bond with 3-hydroxy fatty acids. These are acylated by straight (*n*) and branched (*iso* and *anteiso*) as well as very long-chain fatty acids, i.e., 27-oxooctacosanoic and heptacosanedioic acids, which are rarely encountered in nature (Moll et al., 1992; Zähringer et al., 1995). The outer core oligosaccharide of *L. pneumophila* LPS is a seven-sugar oligosaccharide composed of rhamnose (Rha), mannose (Man), acetylquinovosamine (QuiNAc), and acetylglucosamine (GlcNAc) in the molar ratio of 2.1:1.1:1:1.4 (Knirel et al., 1996; Moll et al., 1997). The outer core oligosaccharide is hydrophobic. Its hydrophobicity is a consequence of the presence of *N*-acetyl groups (QuiNAc and GlcNAc) and methyl groups of 6-deoxy sugars (Rha and QuiNAc). It also results from the substitution of hydroxyl groups at C2 of Rha, C4 of QuiNAc, and C3 of GlcNAc by an acetate residue. The total acetylation of the amino and hydroxyl groups of the oligosaccharide is fully documented for the LPS isolated from *L. pneumophila* serogroup 1 strains isolated from patients (Helbig et al., 1995; Knirel et al., 1996; Kooistra et al., 2002). The inner core, unlike the hydrophobic outer core, is hydrophilic. It contains two 3-deoxy-D-manno-2-octulosonic acid molecules bound by a 2→4 ketosidic linkage and one D-mannose connected to C8 of Kdo present in the inner core. The presence of disaccharide [α-D-Manp(1→8)Kdop] as well as the lack of heptoses and phosphate residues are characteristic of the inner core of *L. pneumophila* LPS (Moll et al., 1997).

The O-antigen-specific chain of the *L. pneumophila* Philadelphia strain is composed of a homopolymer of α-(2-4)-linked 5-acetamidino-7-acetamido-8-*O-*acetyl-3,5,7,9-tetradeoxy-L-glycero-D-galacto-non-2-ulosonic acid, termed legionaminic acid. This unusual sugar lacks free hydroxyl groups and is thus highly hydrophobic, similarly to the outer core oligosaccharide (Knirel et al., 1994).

In the *L. pneumophila* genome, numerous predicted enzymes might contribute to the assembly or modification of LPS, including several acetyltransferases and deacetylases. The *lag-1* gene encoding an *O*-acetyl-transferase transferring the acetyl group to position C-8 of legionaminic acid in the Philadelphia strain is responsible for the reactivity with monoclonal antibody mAb3-1 of the internationally used monoclonal panel for *L. pneumophila* serogroup 1 strain subtyping (Joly et al., 1986; Zou et al., 1999). This LPS modification is largely associated with *L. pneumophila* serogroup 1 clinical disease and predominates in outbreak strains but is less frequently found in environmental *L. pneumophila* serogroup 1 (Helbig et al., 1995; Zou et al., 1999; Doleans et al., 2004). Over 200 clinical *Legionella* isolates were subjected to comparative genome analysis using microarrays. It was found that the LPS biosynthesis gene cluster of serogroup 1 was the only common feature of *L. pneumophila* 1 strains. This suggests that the specific LPS of serogroup 1 is at least partly responsible for the predominance of this serogroup in human disease (Cazalet et al., 2008).

Despite the increasing knowledge of the biology and pathogenicity of this microorganism, it is still unknown why only one species, i.e., *L. pneumophila* serogroup 1, of the over 60 described *Legionella* species is responsible for more than 80% of laboratory confirmed legionellosis cases (Fields et al., 2002; Yu et al., 2002; Beaute et al., 2013). The predominance of *L. pneumophila* serogroup 1 may be in part related to the fact that currently 97% of clinical diagnoses are obtained using a urinary antigen test, which is specific for serogroup 1 (Pierre et al., 2017).

The scope of this work was to demonstrate that the length of the O-specific chain of LPS from *L. pneumophila* serogroup 1 determines the structure of other cell surface components (phospholipids, proteins), which in turn influences the physicochemical properties of the bacterial cell surface and the interaction with the host cell. The analyses of the differences in the structure of surface components between the *L. pneumophila* serogroup 1 wild-type strain and its spontaneous mutant defective in O-specific chain biosynthesis were performed using spectroscopic (NMR, nuclear magnetic resonance and FTIR, fourier transform infrared spectroscopy) and spectrometric (MALDI-TOF MS/MS, matrix-assisted laser desorption/ionization time-of-flight mass spectrometry; GLC/MS, gas-liquid chromatography mass spectrometry) methods. Atomic force microscopy (AFM) was used to check how the differences in the surface components determine the cell surface topography of these bacteria and their nanomechanical properties. Monitoring and analysis of the bacterial interactions with *Acanthamoeba castellanii* cells or THP-1 macrophages were performed with the use of Förster resonance energy transfer.

## Materials and Methods

### Bacterial Strains and Growth Conditions

Two strains were used for the study: *L. pneumophila* serogroup 1 Corby strain (MAb 3/1positive, MAb 26/1-negative) and its spontaneous mutant TF3/1 (MAb 3/1-negative).

The mutant contained a single nucleotide exchange in position 169 in the *lag-1* gene encoding *O-*acetyltransferase, which resulted in a reduced degree of substitution with *O*-acetyl groups in legionaminic acid. The mutant also failed to produce high-molecular-weight long-chain O-polysaccharide (Lück et al., 2001).

*Legionella pneumophila* strains were grown on charcoal yeast extract (BCYE) plates [1% yeast extract, 1% *N*-(2-acetamido)-2-aminoethanesulfonic acid (ACES; pH 6.9), 3.3 mM L-cysteine, 0.33 mM Fe(NO_3_)_3_, 1.5% Bacto agar, 0.2% activated charcoal] at 37°C in a humified atmosphere with 5% CO_2_ for 3 days. Bacteria cultured on the BCYE medium were harvested with 0.5 M NaCl and centrifuged at 8000 × *g* for 20 min. The cell pellets were washed once with 0.5 M NaCl and once with distilled water and lyophilized. Two grams of bacterial mass were obtained from each strain and subjected to isolation of phospholipids and LPSs.

*Legionella pneumophila* strains were inoculated in buffered yeast extract (BYE) broth and grown at 37°C with agitation at 180 rpm for 24 h to an OD_600_ of 1.8.

### Culture of *Acanthamoeba castellanii*

The culture of *A. castellanii* (strain ATCC 30234 free of intracellular endosymbionts] was carried out in Erlenmeyer flask on 100 ml of peptone-yeast extract-glucose (PYG) medium (containing 1.5% proteose peptone, 0.5% yeast extract, 1% glucose, and mineral salts) (Band, 1959) at pH 6.6. The flasks were inoculated with a 3-day old amoeba culture to obtain an initial population of approximately 5 × 10^3^ cells/ml. The number of cells was determined using a Büchner hemocytometer. The culture was incubated on a rotary shaker with an acentric rotation of 3 cm (110 rev/min) at 28°C for 5 days. Amoebae from the early stationary phase of growth were harvested by centrifugation at 300 × *g* for 10 min and washed in amoeba saline prepared after Band (1959).

### THP-1 Cell Cultures

The THP-1 monocytic leukemia cells were purchased from the American Type Culture Collection (ATCC, No TIB-202) and cultured in RPMI 1640 supplemented with 10% heat-inactivated fetal calf serum (FCS), 10 mM HEPES, 2 mM glutamine, 100 IU/mL penicillin, and 100 μg/mL streptomycin at 37°C in a humidified atmosphere of 5% CO_2_. All media and antibiotics were purchased from Sigma-Aldrich (Steinheim, Germany). For the experiments, the THP-1 cells were differentiated into macrophage-like cells as described earlier (Palusinska-Szysz et al., 2014). Briefly, THP-1 cells seeded at a density of 4 × 10^5^ cells/ml in RPMI 1640 supplemented with 10% FCS onto 24-well plastic plates (Nunc, Roskilde, Denmark) were differentiated via 3-day exposure to 50 ng/ml phorbol 12-myristate 13-acetate (PMA, Sigma-Aldrich) (Daigneault et al., 2010). Next, after washing three times, adherent THP-1 cells were cultured in medium without PMA for three consecutive days with daily replacement with fresh medium (without antibiotics). Shortly before the experiment (subsection “Binding Assay”), the cells from extra wells were counted to calculate the number of bacteria used for infection of one THP-1 cell (MOI, multiplicity of infection). For the FLIM (fluorescence life time imaging microscopy) analysis THP-1 cells were cultured on rounded glass slides placed in wells of 6-well plastic plates (Nunc, Roskilde, Denmark) and differentiated according to the above protocol.

### Extraction of Lipids

Lipids were isolated from the bacterial mass of both strains using the Bligh and Dyer (1959) method. Briefly, the bacterial mass was suspended in 150 ml chloroform/methanol (1/2, v/v) and vigorously mixed on a magnetic stirrer for 4 h. Afterward, the suspension was centrifuged for 30 min, 6000 × *g*, 4°C. The organic phase was collected and a new portion of chloroform/methanol (1/2, v/v) was added to the pellet. Lipid extraction was continued for 3 h. After centrifugation (as above), organic phases were pooled and chloroform and water were added to a final methanol/chloroform/water ratio of 2/2/1.8 (v/v/v). After thorough mixing of the reagents and centrifugation for 20 min, 6000 × *g*, 4°C, the lower organic phase was collected and dried using a rotary evaporator. Next, the samples were suspended in a mixture of hexane/isopropanol (3/2, v/v), mixed thoroughly, and centrifuged for 20 min, 6000 × *g*, 4°C. The lipid-containing organic phases were dried under a nitrogen stream and used in further analyses. Approximately 140 mg of lipids, which accounted for 7% of bacterial mass, were isolated from 2 g of the lyophilized Corby strain, and 107 mg of lipids, which constituted approx. 5% of bacterial biomass, were isolated from 2 g of the TF3/1 strain. The chloroform-methanol extractable lipids of both strains were used for MALDI-TOF analyses. Delipidated and dried cell pellets were used for isolation of LPSs.

### Isolation and Purification of Lipopolysaccharides

The delipidated and lyophilized bacterial masses were suspended in 50 mM sodium phosphate buffer (pH 7.0), supplemented with 5 mM EDTA, and digested with lysozyme (6 mg/g dry mass, 4°C, 16 h). Nucleic acids were degraded by treatment with DNase and RNase (0.3 mg/g dry mass, 37°C, 30 min). Cell proteins were digested by incubation with proteinase K (0.3 mg/g dry mass, room temperature, for 18 h, followed by incubation for 10 min at 60°C). The digested cells were extracted three times with 45% phenol/water at 68°C and the separated layers were dialyzed against tap and deionized water (Westphal and Jann, 1965). Pure LPS preparations were obtained by ultracentrifugation (105000 × *g*, 4°C, 4 h). The LPSs were obtained from the phenol phase: 36 mg (1.8%) in the case of the wild type strain and 20 mg (1.0%) in the case of the mutant. The LPSs were used for SDS-PAGE and chemical characterization by NMR spectroscopy.

### SDS Polyacrylamide Gel Electrophoresis (SDS-PAGE) Analysis of Whole Lysed Cells and LPS Isolated From Both Strains

The bacterial masses were rinsed from the BCYE plates with 0.5M NaCl, centrifuged at 8000 × *g* for 10 min, and washed again with 0.5 M NaCl. The lysing mixture containing 1M Tris HCl at pH 6.8, 2% SDS, 4% 2-mercaptoethanol, 10% glycerol, and bromothymol blue was added to the bacterial pellets at a proportion of 150 μl per 50 mg wet weight. The cell lysis was carried out at 100°C for 10 min. After cooling the samples, 10 μl of a proteinase K solution (15 mg/ml, Sigma-Aldrich) was added and the cells were digested for 3 h at 37°C and next overnight at room temperature. Next, the samples were heated for 1 h at 60°C and for 10 min at 100°C (Apicella et al., 1994). After short spinning, samples prepared in this way were transferred onto polyacrylamide gel.

The LPS preparations were suspended in a sample buffer containing 175 mM Tris–HCl at pH 6.8, 0.25% SDS, 2% glycerol, and 0.04% bromophenol blue and mounted on polyacrylamide gel.

Electrophoretic profiles of lysed cells and LPS from each strain were determined by running the samples in SDS-PAGE. Commercially available LPS (*Salmonella enterica* sv. Thyphimurium, Sigma-Aldrich) was used as a standard for determination of the LPS size. The bands were visualized by silver staining after oxidation with periodate according to the method proposed by Tsai and Frasch (1982).

### Preparation of Fatty Acid Methyl Esters (FAMEs)

Lyophilized bacterial mass of the *L. pneumophila* Corby strain and its mutant (approx. 3 mg) was saponified with 0.8M NaOH in 50% methanol at 80°C for 60 min. Acidification with 6M HCl was followed by extraction of released fatty acids with a chloroform/water mixture (1/2 v/v). After drying the organic phase under a nitrogen stream, the free fatty acids were converted into methyl esters by methanolysis (0.5M HCl in methanol, 85°C, 1.5 h) (Palusinska-Szysz et al., 2016). The solution was cooled to room temperature and evaporated. The recovery of fatty acid methyl esters (FAMEs) was performed by extraction with chloroform/water (1/2 v/v). After centrifugation (5000 × *g*, 10 min), the lower layer of the organic phase was transferred to a glass vial. The extraction was performed twice. After pooling, the organic phases were evaporated to dryness under a nitrogen stream at room temperature. FAMEs were dissolved in 30 μl of chloroform and analyzed by gas-liquid chromatography. All experiments were performed in three independent replicates to evaluate the reproducibility of the data.

### GLC-MS Acquisition and Data Analysis

Gas-liquid chromatography mass spectrometry analyses of fatty acids were performed on an Agilent Technologies chromatograph 7890A connected to a mass selective detector EI/CI MSD 5975C. The chromatograph was equipped with a HP-5MS column (30 m × 0.25 mm). Helium was a carrier gas (flow rate: 1 ml min^–1^). The temperature program was as follows: 150°C for 3 min, then raised to 250°C at 3°C min^–1^ and then to 320°C at 25°C min^–1^. The final temperature was maintained for 20 min. Fatty acids were identified by their characteristic electron impact (EI) fragmentation patterns and retention times. The relative area percentage of the fatty acids was reported.

### FTIR Spectroscopy

Infrared absorption spectra were recorded with a Nicolet 6700 FT-IR spectrometer (Thermo Fisher Scientific, Waltham, MA, United States) with the Smart *iTR* ATR sampling accessory. Approximately 2 mg of bacterial lyophilizates were deposited on ATR crystal. Absorption spectra were collected in the region between 4000 and 650 cm^–1^. For each bacterial lyophilizate, three samples were examined in the same conditions. For each sample, 200 scans were averaged with a spectral resolution of 4 cm^–1^. Then, a final average spectrum was calculated for each sample. Baseline corrections if needed and normalization were obtained using Omnic Software (v. 8.2, Thermo Fisher Scientific Inc., United States). Further analysis of spectra was performed using Origin Software (OriginLab v8.5 Pro, Northampton, United States).

### MALDI-TOF MS and MS/MS Spectrometry

Matrix-assisted laser desorption/ionization time-of-flight mass spectrometry (MALDI-TOF–MS) was performed using a Waters SYNAPT G2-Si HDMS instrument (Waters Corporation, Milford, MA, United States) equipped with a 1 kHz Nd:YAG laser. Phospholipids isolated from the wild-type cells and the mutant cells were dissolved in chloroform/methanol (2/1, v/v) at a concentration of 10 mg mL^–1^. One microliter of the sample was mixed with one microliter of the matrix, transferred into the target plate, and allowed to dry. The matrix solution was prepared from 2,5-dihydroxybenzoic acid (DHB, Sigma-Aldrich) (10 mg mL^–1^, chloroform/methanol 2/1, v/v). Spectra were recorded in positive and negative ion modes. Data acquisition was performed using MassLynx software version 4.1 SCN916 (Waters Corporation, Wilmslow, United Kingdom). For the MS/MS experiments, the isolated precursor ions were fragmented using collision voltage of 35 V for PC, PE, PG, and 60 V for CL. Data were collected for 120 s for each ion separately. Mass spectra were assigned with a multi-point external calibration using red phosphorous (Sigma-Aldrich). The regiochemistry of phospholipids was determined based on the intensity ratio of *sn-2/sn-1* carboxylate anions. This ratio was higher than 1 for PC, PG, and PE (Fang and Barcelona, 1998).

### NMR Spectroscopy

High-resolution magic angle spinning (HR-MAS) NMR experiments were performed on a Bruker Avance III 400 spectrometer (operating frequencies 400.13 MHz for ^1^H NMR) equipped with a 4 mm HR MAS probe and ZrO2 rotors. The samples were re-solubilized in a H_2_O/D_2_O (9:1) mixture. The other NMR parameters were as follows: MAS rotation rate 4.5 kHz, spectral range 8012 kHz, acquisition time 2 s, line-broadening (LB) of 1 Hz, number of scans 512. Spectra were collected with the original Bruker pulse program employing watergate W5 pulse sequence with gradients using double echo. The spectral data were processed using the TopSpin 2.1 program (Bruker) operating in the Windows XP Professional environment.

### AFM

A total of 40 microliters of the *L. pneumophila* (wild type and mutant strain separately) suspension (OD_600__nm_ = 0.2) prepared from bacteria grown for 3 days on BCYE plates were aliquoted to three Eppendorf tubes each. After washing three times in MQ water, centrifugation (8000 × *g*, 10 min, 4°C), and pooling, the bacteria were suspended in 5 μl of non-pyrogenic water and mounted on the surface of freshly cleaved mica discs for imaging.

The *Legionella pneumophila* cell surface structure and nanomechanical properties (adhesion) were imaged and analyzed using NanoScope V AFM (Veeco, United States). All measurements were performed in the “ScanAsyst-HR” operation mode using a silicon tip with a spring constant of 0.4 N/m (SCANASYST-AIR-HR, Bruker, Germany). The data were analyzed with Nanoscope Analysis ver. 1.40 software (Veeco, United States) (Analytical Laboratory, Faculty of Chemistry, Maria Curie-Skłodowska University, Lublin, Poland). Three fields were imaged on each mica disc. The resolution of the scans obtained was 384 × 384 pixels. The topography of the examined samples was presented as height and peak force error images.

### Electron Microscopy

The bacterial cells were gently scraped from the BCYE agar surface and collected into the vials containing 2% formaldehyde (freshly prepared from paraformaldehyde) and 2.5% glutaraldehyde dissolved in 0.1 M sodium cacodylate buffer (pH 7.4). The bacterial cells were preliminarily fixed in this way. After 5 min, the cell suspension was centrifuged at 1000 × *g* for 10 min. The cell pellets were fixed for 4 h at 4°C in buffered glutaraldehyde and formaldehyde (2.5% and 2%, respectively). After rinsing three times with 0.1 M cacodylate buffer (pH 7.4), the cell pellets were post-fixed in a 1% osmium tetroxide solution in 0.1 M sodium cacodylate buffer (pH 7.4) for 20 min at 4°C. After rinsing three times with 0.1 M cacodylate buffer (pH 7.4), 40 μl of 2% agar at temp. 45°C were added to the cell pellets. After thorough mixing, the agar-suspended bacteria were transferred to slides and the gelled material was cut into small fragments.

The bacterial cells were dehydrated in a series of alcohol (30%, 50%, 70%, and 90%) and twice in 100% alcohol and embedded in LR White resin. Blocks were polymerized in an incubator at 55°C. Ultrathin sections (80 nm) were cut with a diamond knife on a microtome RMC MT-XL (Boeckeler Instruments, Tucson, AZ, United States) collected on copper grids and contrasted with the use of uranyl acetate and Reynold’s liquid. The samples were observed under a LEO-Zeiss 912-AB electron microscope (Carl Zeiss Microscopy, Oberkochen, Germany). Morphometric measurements were conducted with iTEM Soft Imaging Solutions software dedicated to the transmission electron microscopy camera.

### Binding Assay

Binding assays were performed with THP-1-derived macrophages and *A. castellanii* cells plated at 2 × 10^5^ cells/well in 24 well plates. The cell monolayers were pre-incubated with cytochalasin D (1 μg/ml, Sigma-Aldrich) for 2 h at 35°C to prevent phagocytosis. Afterward, the *L*. *pneumophila* strains were added to the THP-1-derived macrophages (multiplicity of infection MOI = 20) and *A. castellanii* (MOI = 50). The infections were synchronized by centrifugation at 300 × *g* for 5 min. After 30 min of co-incubation of the bacteria with the macrophages and the bacteria with the amoeba at 35°C, the monolayers were washed three-times with PBS to remove non-adherent bacteria. Appropriate 10-fold dilutions of the inoculum and bacteria recovered from cells lysed with distilled water were plated on BCYE agar and incubated at 37°C for up to 4 days before *L. pneumophila* colony-forming units (CFU) were counted. The results were expressed as a ratio of adherent bacteria compared to the inoculum.

### FRET Assay

For the FLIM analysis, *L. pneumophila* cells (Corby strain and TF3/1 strain) (100 μl of suspension each, OD_600_ = 0.2) in MQ water were incubated with 5 μM SYTO9 green fluorescence stain in 50 mM Tris pH 7.0 buffer at 23°C for up to 20 min (Thermo Fisher Scientific, United States). The bacterial suspensions were centrifuged at 5000 × *g* for 10 min at RT. The cells were gently washed four times with non-pyrogenic water (250 μl) and finally suspended in 100 μl of non-pyrogenic water. Then, 10 μl of the suspensions were applied on a polylysine-coated cover slip and analyzed immediately using FLIM microscopy.

The kinetics of the interaction between *L. pneumophila* (Corby strain and TF3/1 strain) and *A*. *castellanii* or THP-1 macrophages was determined by FRET spectroscopy with the use of a MicroTime 200 microscopic confocal fluorescence system (PicoQuant GmbH, Germany). SYTO9-labeled *L. pneumophila* strains (Corby and TF3/1) were added to the *A. castellanii* cells or THP-1 macrophages (labeled with Nile Blue) in a dose of MOI 50 or 20 MOI. The samples were placed on special non-fluorescing coverslips (Menzel-Gläser) coated with polylysine, and analyzed immediately. Pulsed excitation laser beams (470 nm for the donor and 635 nm for the acceptor) were guided to the sample by a dichroic mirror (ZT470/488/640/RPC) and further by a high numerical aperture objective 60× (NA 1.2). Emission from excited molecules was collected and focused onto a 50-μm diameter pinhole to facilitate confocal detection. The fluorescence signal coming from a set of samples labeled a donor (SYTO9), an acceptor (Nile Blue, NB), and a donor-acceptor pair was separated by a dichroic beam splitter (620 dcxxr) and filtered spectrally by narrow band clean-up filters (520/35 for the donor) and (690/70 for the acceptor) placed separately in front of two identical avalanche photo detectors (τ-SPAD). All the dichroics and filters were purchased from Analysentechnik (Germany). The lasers working in a pulse-interleaved mode were electronically delayed with respect to each other by 50 ns. The excitation with the red laser yielded fluorescence from the acceptor molecule, while the excitation of the donor molecule with the 470 nm laser resulted in emission from the donor and from the acceptor molecule due to FRET. Photon detection was accomplished in the time-correlated single photon counting mode with the TimeHarp 400 board. The time resolution was set to 16 ps. The data were analyzed with the SymPhoTime (v. 2.3) software package (PicoQuant, Geramany). The intensity decays *I(t)* for each sample were deconvoluted with a multi-exponential model:

$$I\,\left(t\right)=\sum_{i}\alpha_{i}\,e\,x\,p\,\left(-\frac{t}{\tau_{i}}\right)$$

where *τ_*i*_* are the characteristic decay times and α*_*i*_* are the pre-exponential factors. The average lifetime was calculated based on the amplitude-weighted formula:

$$\left\langle \tau \right\rangle =\frac{\sum_{i}\alpha_{i}\,\tau_{i}}{\sum_{i}\alpha_{i}}$$

used further to calculate an average energy transfer efficiency *E*_*T*_ according to the formula:

$$E_{T}=1-\frac{\left\langle \tau_{D\,A}\right\rangle }{\left\langle \tau_{D}\right\rangle }$$

where <*τ_*DA*_*>and <*τ_*D*_*> are amplitude weighted average donor lifetimes in the presence and absence of the acceptor, respectively. The efficiency of the energy transfer was calculated for all image pixels and presented as a distribution on appropriate histograms and color-coded pictures.

The donor-acceptor distance was calculated based on Förster distance (*R*_0_) expressed with following formula (Lakowicz, 2010):

$$R_{0}=8.79\times 10^{3}\,{\left[Q_{D}\,\kappa^{2}\,n^{-4}\,J\,\left(\lambda \right)\right]}^{-\frac{1}{6}}$$

where *Q*_*D*_ is the fluorescence quantum yield of the donor in the absence of the acceptor (*Q_*D*_* = 0.58 for SYTO9 in physiological fluid), κ*^2^* is an orientation factor (here 2/3), *n* is the refractive index of the medium (*n* = 1.33), and *J(*λ) is the overlap integral expressed in *M*^–^*^1^cm^3^* units:

$$J\,\left(\lambda \right)=\frac{\int F_{D}\,\left(\lambda \right)\,\varepsilon_{A}\,\left(\lambda \right)\,\lambda^{4}\,d\,\lambda }{\int F_{D}\,\left(\lambda \right)\,d\,\lambda }$$

*F_*D*_(*λ) denotes donor fluorescence intensity, ε*_*A*_(*λ) is the extinction coefficient of the acceptor expressed in M^–1^cm^–1^ units, and λ is the wavelength in *cm* units. Based on the steady-state spectra and taking into account the molar decadic extinction coefficient for NB 18000 M^–1^cm^–1^, the Förster distance (*R*_0_) of SYTO9-NB donor-acceptor pair is 39.5 Å.

### Statistical Analysis

Values were expressed as mean ± SD from three independent experiments (adhesion of *L. pneumophila* to differentiated THP-1 cells and *A. castellanii*). The results were statistically evaluated using the Mann-Whitney *U* test (STATISTICA software version 7.1, StatSoft Inc., Tulsa, OK, United States). *P* values of ≤0.05 were considered significant.

## Results

### FTIR Analysis of *L. pneumophila* Cells

**FIGURE 1:**
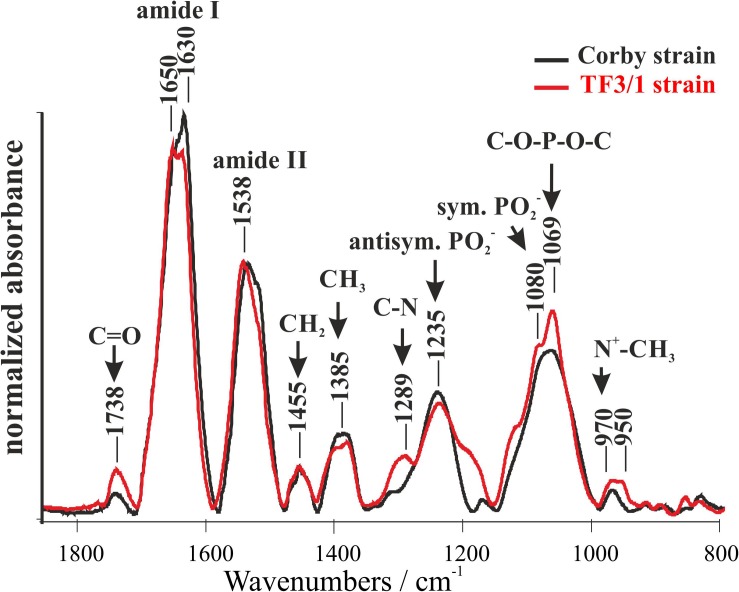
Fourier-transform infrared spectroscopy (FTIR) spectra of the *Legionella pneumophila* Corby strain and the TF3/1 mutant. The spectra were normalized vs. the 1455 cm^–1^ band.

FTIR spectroscopy was used to analyze the components of *L. pneumophila* cells (Corby strain and TF3/1 strain). This technique facilitates recording infrared absorption spectra that reflect vibrations of chemical bonds and specific groups of molecules. Several relatively intensive IR absorption bands can be visible in the whole bacterial spectra (Figure 1). The band between 950 and 970 cm^–1^ can be assigned to the antisymmetric stretching vibrations of N^+^–CH_3_ groups (assigned to choline in phosphatidylcholine). This band shows differences between the strains. The *L. pneumophila* Corby strain contained fewer N^+^–CH_3_ groups than the mutant strain. The band centered at 1069 cm^–1^ represents the stretching skeletal vibrations of the C-O-P-O-C group in polar phospholipid heads. The bands at 1080 cm^–1^ and 1235 cm^–1^ represent the symmetric and antisymmetric stretching of the PO^2–^ groups, respectively. The band centered at 1385 cm^–1^ can be assigned to the deformation “umbrella” vibrations in the CH_3_ groups and the band centered at 1455 cm^–1^ to the deformation “scissoring” vibrations in the CH_2_ groups. The prominent bands at 1500–1600 cm^–1^ and at 1600–1700 cm^–1^ represent amide II and amide I group vibrations, respectively. The spectral component of the amide I band centered at 1650 cm^–1^ represents α-helical structures since the component at 1630 cm^–1^ represents parallel β-structures (Tamm and Tatulian, 1997). The higher intensity of the 1630 cm^–1^ band in the Corby strain can be interpreted in terms of higher abundance of β structures (Tamm and Tatulian, 1997). It may also indicate a higher level of protein aggregation (Tamm and Tatulian, 1997). On the other hand, it is highly probable that the higher intensity in the spectral region of 1630 cm^–1^ in the Corby strain spectrum is associated with longer O-specific chains, due to the contribution of C = N stretching vibrations in the legionaminic acid. The band at 1738 cm^–1^ represents C = O stretching of ester carbonyl groups in acyl lipids and in LPS fragments. The higher intensity of this band observed in the case of the TF3/1 strain can be interpreted in terms of the higher concentration of those groups, relative to the number of CH_2_ groups (owing to the fact that the spectra were normalized at the band representing methylene group vibrations at 1455 cm^–1^). The two bands, centered at 1289 cm^–1^ and in the region of 1200 cm^–1^, particularly intensive in the case of the TF3/1 strain, can be assigned to the C-N stretching vibrations in nucleic acids and aromatic amines.

### SDS Polyacrylamide Gel Electrophoresis (SDS-PAGE) Analysis of Whole Cells and LPS of Both Strains

**FIGURE 2:**
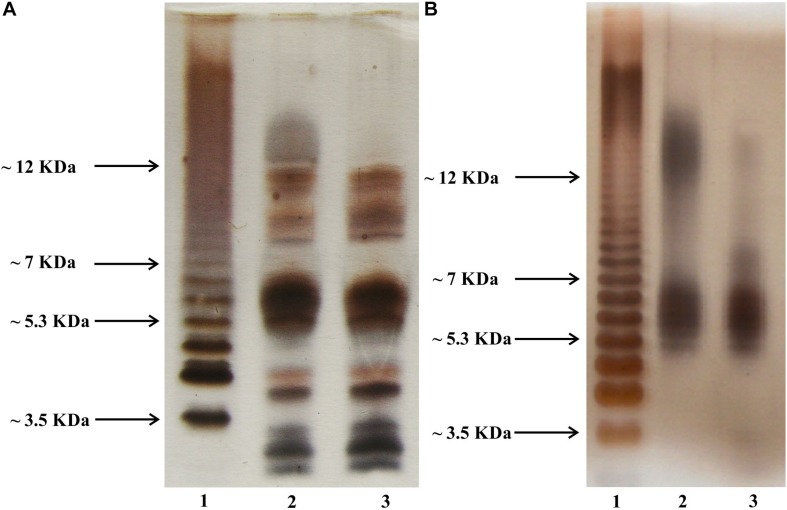
Silver-stained SDS PAGE of whole bacteria **(A)** and phenol-soluble LPS fraction **(B)**. Line 2, *L. pneumophila* Corby strain; Line 3, TF3/1 strain; Line 1, *Salmonella enterica* sv. Typhimurium LPS reference.

The SDS-PAGE profiles of *L. pneumophila* LPS as well as the SDS-PAGE profiles of whole bacteria digested by proteinase K according to Apicella and co-workers (Apicella et al., 1994) revealed substantial similarities in the mass range below 12 kDa (Figures 2A,B, lines 2 and 3). Practically, in this mass range, each band on the electropherogram of the LPS of the preparation obtained by digestion of the TF3/1 bacterial mass with proteinase K had its counterpart in the LPS preparation from the parental strain (*L. pneumophila* Corby). This was very similar in the case of the electrophoretic separation of purified LPS preparations from both tested strains. The absence of fast migrating bands (below 5 kDa) in the case of LPS preparations can be explained by the possible washing out during the delipidation of bacterial masses. This assumption is quite likely due to the strongly hydrophobic character of the lipid A core molecules (Kooistra et al., 2001). A substantial difference in the length of the polysaccharide LPS chains between the *L. pneumophila* Corby strain and its mutant was visible near and above 12 kDa. The mutant did not produce or produced only sparse amounts of a high-molecular-weight fraction of LPS (so called smooth-LPS) (Figures 2A,B, lines 2 and 3).

### NMR Spectroscopy

**FIGURE 3:**
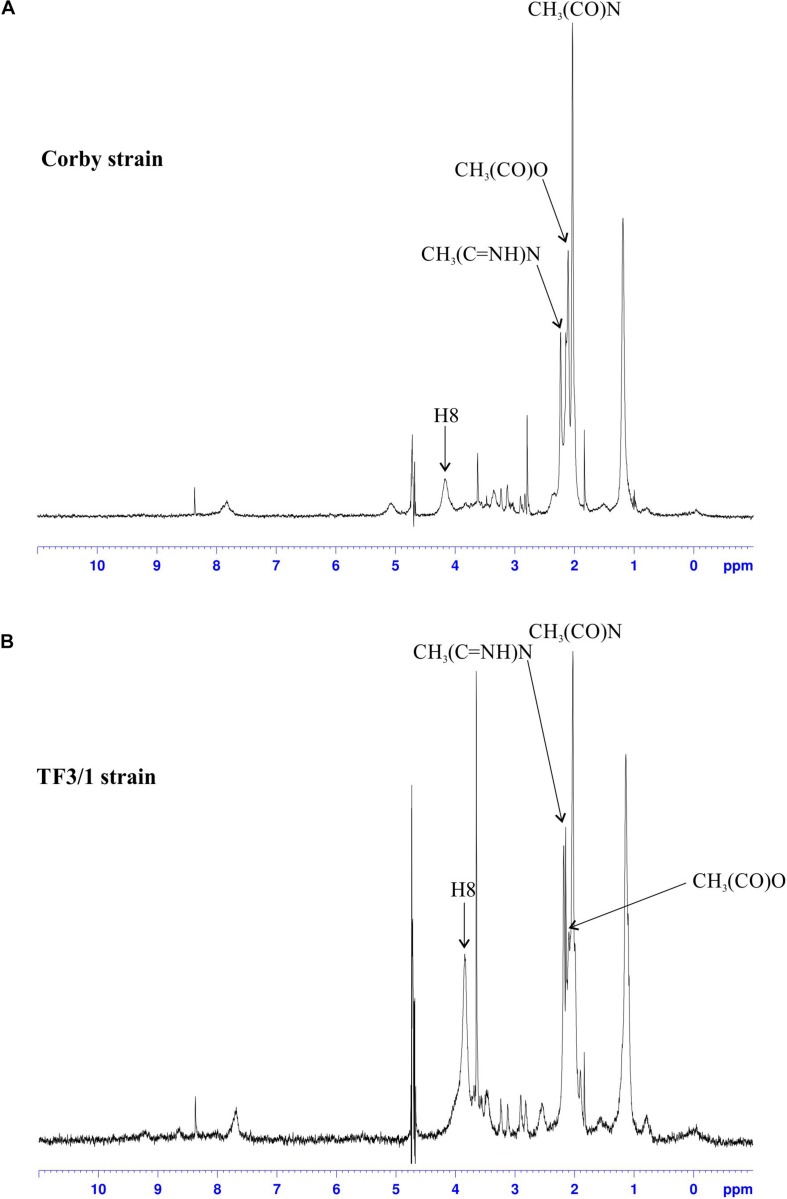
^1^H NMR spectra of the LPS from the Corby strain **(A)** and the TF3/1 strain **(B)**. Only the signals from *O*-acetyl, *N*-acetyl and *N*-acetimidoyl groups as well as from the H8 proton of legionaminic acid are marked on the spectra.

The difference in the content of *O*-acetyl residues in the legionaminic acid of LPS between the *L. pneumophila* Corby strain and its mutant was shown using ^1^H HR-MAS NMR (Figures 3A,B). The TF3/1 strain cannot produce the 8*-O*-acyltransferase of legionaminic acid due to the point mutation in the *lag-1* gene. The lack of the enzymatic activity of the Lag-1 protein is documented in the spectra. The analysis of the region of occurrence of signals of the *O*-acetyl and *N*-acetyl and *N*-acetimidoyl methyl groups (2.00–2.30 ppm) on the spectrum of LPS isolated from the Corby strain revealed a clear signal from CH_3_(CO)N protons, two signals from CH_3_(C = NH)N protons, and a CH_3_ signal of the *O*-acetyl residues between them (δ 2.11 ppm). The last one almost disappeared from the LPS TF3/1 spectrum.

Moreover, the signals from H8, H7, H4, and H6 (merging on the Corby spectrum into one broad peak) were shifted by 0.3 ppm (from ∼4.2 to ∼3.9) (Figure 3). Both strains produced *O*-acetyl substituents in the LPS, but the mutant type produced significantly less *O*-acetyl residues than the Corby strain (Kooistra et al., 2001).

### Fatty Acid Composition

The analysis of fatty acid methyl esters (FAMEs) showed the presence of 31 different acids from 14 to 22 carbon atom in hydrocarbon chains, including straight saturated and unsaturated fatty acids as well as branched ones (*iso* and *anteiso*) (Table 1). Both analyzed strains were characterized by high content of branched fatty acids (Corby strain 58 mol%, mutant 47 mol%). i16:0 (28 mol% in the wild type strain, 24 mol% in the mutant) and *a*15:0 were the major branched acids. Cyclopropane 17:0cyc was also found in both strains. The comparative analysis of the FA composition showed quantitative differences between the *L. pneumophila* Corby strain and its mutant.

**TABLE 1:** The fatty acid composition of *L. pneumophila* Corby and TF3/1 strain cells.

**Retention time**	**Fatty acid**	**Content (given in mol%)**	
		**Corby strain**	**TF3/1 strain**
10.39	*i*14:0	4.5 ± 0.2	4 ± 0.4
10.95	14:1	tr	tr
11.20	*n*14:0	1 ± 0.1	1 ± 0.2
12.56	*i*15:0	0.5 ± 0.2	tr
12.72	*a*15:0	15 ± 0.6	11 ± 0.5
13.01	15:1	1 ± 0.1	1 ± 0.4
13.36	*n*15:0	1 ± 0.2	1 ± 0.4
14.27	*i*16:1	1 ± 0.3	1 ± 0.3
14.69	*i*16:0	28 ± 1	24 ± 0.5
15.02	*n*16:1	14 ± 0.5	16 ± 0.2
15.43	*n*16:0	8.5 ± 0.2	8 ± 0.5
15.55	*i*17:1	tr	tr
16.42	*n*17:1	tr	tr
16.68	*i*17:0	tr	tr
16.85	*a*17:0	8 ± 0.2	7 ± 0.5
17.13	17:0*cyc*	4 ± 0.1	4 ± 0.2
17.41	*n*17:0	1 ± 0.2	1 ± 0.5
18.60	*a*18:0	tr	tr
19.28	*n*18:0	4.5 ± 0.4	5 ± 0.5
20.44	*i*19:0	tr	tr
20.60	*a*19:0	tr	tr
21.09	*n*19:0	1 ± 0.3	1 ± 0.6
22.18	*i*20:0	tr	tr
22.79	*n*20:0	5 ± 0.5	12 ± 0.6^∗^
23.84	*i*21:0	tr	tr
24.02	*a*21:0	tr	tr
24.46	*n*21:0	1 ± 0.1	1 ± 0.2
25.46	*i*22:0	tr	tr
25.63	22:1	tr	1 ± 0.5
25.67	*a*22:0	1 ± 0.2	tr
26.05	*n*22:0	tr	1 ± 0.4
Sum of branched		58	47

**a*, methyl branch at the *anteiso* carbon atom; *i*, methyl branch at the *iso* carbon atom; *n*, unbranched acid, *cyc*, cyclopropane ring structure; tr, trace, amounts below 0.5%. ^∗^The difference was statistically significant at *P* ≤ 0.028.*

The Corby strain synthesized more branched FAs (*a*15:0, *i*16:0, and *a*17:0) as well as less unsaturated 16:1 and straight chain 18:0 acids than the mutant. In turn, the mutant synthesized approximately twofold higher amounts of long-chain fatty acid (20:0) than the wild type. This difference was statistically significant at *P* ≤ 0.028.

### MALDI-TOF Mass Spectrometry

The phospholipid structure in the *L. pneumophila* Corby strain and the TF3/1 strain were analyzed with MALDI-TOF mass spectrometry. The positive ionization MALDI-TOF spectrum indicative of the molecular masses of the individual phospholipids showed a clear cluster, which represented mainly phosphatidylcholines (PC) (at m/z 600.40–850.48) (spectrum not shown). In the negative ionization MALDI-TOF spectrum, there were two clusters of peaks: one represented phosphatidylethanolamines (PE) and phosphatidylglycerols (PG) (m/z 600.50–800.45) and the other corresponded to cardiolipins (CL) (at m/z 300.40–1450.45) (spectrum not shown).

The MS/MS experiments were performed by inducing fragmentation on sodiated precursor ions derived from PCs in the positive ionization mode. The spectra showed two diagnostic ions for the choline head group: a significant fragment ion at m/z 184.15 corresponding to cholinephosphate [C_5_H_13_NPO_4_]^+^ and a less intense m/z ion 147.07 [C_2_H_5_O_4_P + Na]^+^. These spectra contained fragment ions: m/z ions, ions resulting from the neutral loss of trimethylamine (59u), m/z ions resulting from the neutral loss of cholinephosphate (183u), and ions corresponding to the loss of sodiated cholinephosphate (205u). Furthermore, there were less abundant ions relevant to the loss of fatty acid substituents such as ions corresponding to [M-59-fatty acyl + Na]^+^ and [M-59-fatty acyl + H]^+^.

The fatty acid distributions of asymmetrical PEs and PGs were assigned using negative ionization mode MALDI-TOF MS spectra. Localizations of the fatty acids on the *sn* positions were determined based on the ratio of the intensity of fragment ions representing each fatty acid, which were prominent on these spectra (Fang and Barcelona, 1998). The PE spectra contained intensive ions resulting from [HPO_4_CH_2_CH_2_NH_2_ + ketene]^–^ and less intensive [M-H-ketene]^–_^derived ions. The diagnostic ion on the PG spectra was located at m/z 171. Detailed information on the structure of *L. pneumophila* phospholipids is provided in Tables 2, 3.

**TABLE 2:** The major molecular species of *L. pneumophila* Corby and TF3/1strain phospholipids by MALDI-TOF MS/MS operating in the negative (−) and the positive (+) ion mode.

**Phospholipid class**	**m/z**	***sn-1/sn-2***	
		**Corby**	**TF3/1**
PC	728.44 (+)	tr	16:0/14:0^∗^
	742.45 (+)	16:0/15:0, 17:0/14:0^∗^	16:0/15:0, 17:0/14:0^∗^
	756.45 (+)	16:0/16:0, 17:0/15:0, 18:0/14:0^∗^	16:0/16:0, 17:0/15:0, 18:0/14:0^∗^
	768.45 (+)	16:0/c17:0,16:0/17:1^∗^	16:0/17cyc:0,16:0/17:1^∗^
	782.46 (+)	18:0/16:1,17:0/17:0cyc, 17:0/17:1^∗^	17:0/17:0cyc,17:0/17:1^∗^
	810.48 (+)	–	20:0/16:1^∗^
PE	674.92 (−)	16:1/15:0, 17:0/14:1, 17:0cyc/14:0, 15:1/16:0	16:1/15:0, 16:0/15:1, 14:0/17:0cyc,17:0/14:1
	702.90 (−)	17:0cyc/16:0,17:1/16:0, 17:0/16:1, 18:0/15:1	17:0cyc/16:0,17:1/16:0, 17:0/16:1
	704.92 (−)	16:0/17:0cyc, 18:0/15:0, 19:0/14:0	16:0/17:0,18:0/15:0,19:0/14:0
	718.83 (−)	17:0/17:0, 19:0/15:0	17:0/17:0, 19:0/15:0
	730.92 (−)	19:0/16:1	19:0/16:1
	732.90 (−)	19:0/16:0	19:0/16:0
	744.93 (−)	20:0/16:1	20:0/16:1
	758.76 (−)	20:0/17:0cyc, 21:0/16:1	tr
	760.90 (−)	20:0/17:0	20:0/17:0
	774.76 (−)	20:0/18:0, 21:0/17:0	tr
PG	691.90 (−)	tr	16:1/14:0
	719.83 (−)	–	16:1/16:0
	735.15 (−)	18:0/15:0, 17:0/16:0, 19:0/14:0	16:0/17:0
	753.15 (−)	20:1/14:1, 16:1/18:1, 17:0cyc/17:0cyc,17:1/17:1	–
	761.76 (−)	16:1/19:0, 21:0/14:1	tr
	763.76 (−)	–	20:0/15:0, 19:0/16:0

*c, cyclopropane ring structure, ^∗^Fatty acids without established *sn-1/sn-2* position; tr, trace; PC, phosphatidylcholine; PE, phosphatidylethanolamine; PG, phosphatidylglycerol. For phosphatidylcholines the pseudomolecular ion is their sodium adduct. Any 17:1 fatty residue denotes either the cyclopropane 17:0 or unsaturated 17:1 fatty acid (indistinguishable under the present mass spectrometry conditions).*

**TABLE 3:** Survey of molecular species of *L. pneumophila* Corby strain and TF3/1 strain cardiolipins determined by MALDI-TOF MS/MS operating in the positive (+) ion mode.

**m/z**	**Fatty Acids (^∗^)**	
	**Corby**	**TF3/1**
1317 (+)	14:0/16:1/16:1/16:1	14:0/16:1/16:1/16:1
	14:0/15:1/16:1/17:0cyc or 17:1	14:0/15:1/16:1/17:0cyc or 17:1
	15:0/15:1/15:1/17:0cyc or 17:1	15:0/15:1/15:1/17:0cyc or 17:1
	15:0/15:1/16:1/16:1	15:0/15:1/16:1/16:1
		14:1/15:1/15:1/18:0
		15:1/15:1/15:1/17:0
1331 (+)	15:0/16:1/16:1/16:1	15:0/16:1/16:1/16:1
	15:1/15:1/16:1/17:0	15:1/16:0/16:1/16:1
	14:0/16:1/16:1/17:0cyc or 17:1	14:0/16:1/16:1/17:0cyc or 17:1
		15:1/15:1/15:1/18:0
1343 (+)	–	15:1/16:1/16:1/17:0cyc or 17:1
		15:1/16:1/16:1/17:0cyc or 17:1
		15:1/15:1/17:0cyc/17:0cyc or 17:1
1345 (+)	16:1/16:1/16:1/16:0	–
	14:1/15:1/15:1/20:0	
	15:1/16:1/16:1/17:0	
1357 (+)	16:1/16:1/16:1/17:0cyc or 17:1	16:1/16:1/16:1/17:0cyc or 17:1
	15:1/16:1/17:0cyc/17:0cyc or 17:1	15:1/16:1/17:0cyc/17:0cyc or 17:1
1371 (+)	16:1/16:1/17:0cyc/17:0cyc or 17:1	16:1/16:1/17:0cyc/17:0cyc or 17:1
1385 (+)	16:1/17:0cyc/17:0cyc/17:0cyc or 17:1	16:1/17:0cyc/17:0cyc/17:0cyc or 17:1
1399 (+)	–	17:0cyc/17:0cyc/17:0cyc/17:0cyc or 17:1
1429 (+)	16:1/17:0cyc/17:0cyc/20:0 or 17:1	–

*Cyc, cyclopropane ring structure. 17:0cyc and C17:1 is indistinguishable in this method. ^∗^Fatty acids without established *sn-1*/*sn-2*/*sn-3*/*sn-4* position.*

The main differences in the phospholipids between both strains were found in the classes of PCs and PGs. In the PC class, the mutant type produced PC with molecular weight 810.48u and a fatty acid distribution of 20:0/16:1. In turn, in the PG class, the wild type strain produced PG with molecular weight 753.15u and a fatty acid distribution of 20:1/14:1, 16:1/18:1, 17:0cyc/17:0cyc, and 17:1/17:1. The TF3/1 mutant produced PG with molecular mass 719.83u and with fatty acid distribution of 16:1/16:0 as well as PG with molecular mass 763.76u and with fatty acid distribution of 20:0/15:0, 19:0/16:0.

### Transmission Electron Microscopy

**FIGURE 4:**
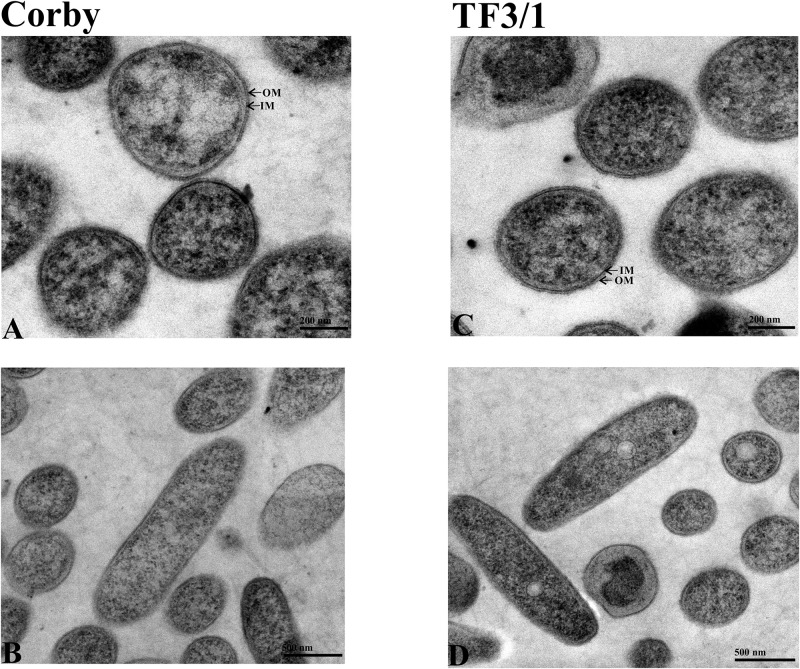
TEM images of cells of *L. pneumophila* Corby strain **(A,B)** and TF3/1 strain **(C,D)**. **(A,C)** Enlarged view of cells showing a well visible arrangement of the cell envelope containing the outer membrane (OM) and inner membrane (IM) (arrows). **(B,D)** Cells in the longitudinal section with granular cytoplasm.

Microscopic observations of both strains revealed the presence of small vacuoles, a well discernible outer and inner membrane, and ribosome-containing cytoplasm. The cells had a longitudinal shape. Seventy cells of each strain were measured. The bacilli were usually 1.5 to 2.0 μm in length and 0.4 to 0.5 μm in diameter. The cell wall thickness was 14.96 ± 1.59 nm in the Corby strain and 14.48 ± 1.36 nm in the TF3/1 strain, and the differences were not statistically significant (Figure 4).

### Atomic Force Microscopy

**FIGURE 5:**
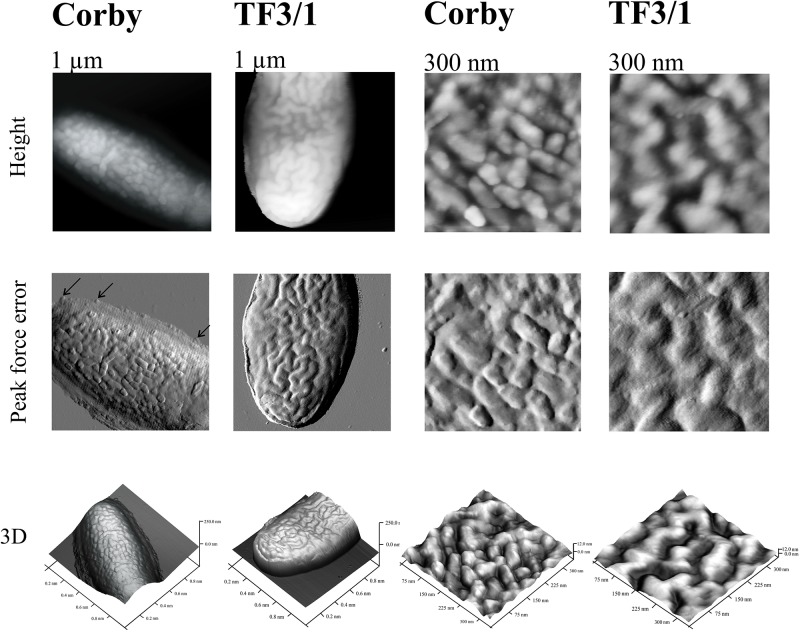
Cells of *L. pneumophila* Corby strain and TF3/1 strain imaged by AFM. The height, “peak force error,” and three-dimensional (3D) images are presented. The **right** and **left panels** demonstrate imaged areas of 1 μm × 1 μm and 300 nm × 300 nm, respectively. Arrows indicate detached vesicles.

Atomic force microscopy was used to check how the differences in the surface components determine the cell surface topography of these bacteria and their nanomechanical properties. The height image showed that the Corby strain had no undulated surface and produced numerous vesicles, which in fact covered the whole surface. Vesicles detached from the bacteria were visible as well. In the case of the mutant type, there were not as many vesicles, but grooves were visible (groove depth 27.5 nm) and the bacteria were distinctly separated from the surroundings (Figure 5). The section analysis carried out in the height image showed that the height of the Corby strain bacterium from the substrate to the apex was 137 nm. The analysis of surface morphology demonstrated average roughness of the cell surface of 1.7 nm and the diameter of the vesicles of 45 nm ± 7 nm. The average roughness of strain TF3/1 was 3.3 nm and differed from that of the Corby stain. There were distinct differences in the adhesive properties between the strains. The Corby strain exhibited higher mean values of adhesion than the mutant (mean Corby strain adhesion – 2.39 ± 0.15 nN; mean mutant type adhesion – 1.59 ± 0.11 nN). In the case of the mutant type, the adhesion was not the same over the whole cell surface and there were areas with greater or lower adhesion due to surface undulation.

### Lipopolysaccharide Contributes to Host Cell Adherence

#### Binding Assay

To compare the adhesive affinity of the *L. pneumophila* Corby strain and the TF3/1 strain, phagocytosis was prevented with the actin polymerization inhibitor cytochalasin D. The binding assay revealed that the adherence of the TF3/1 cells to macrophages and *A. castellanii* was reduced by 18% and 15%, respectively, compared to the *L. pneumophila* Corby strain.

#### FLIM-FRET Measurements

**FIGURE 6:**
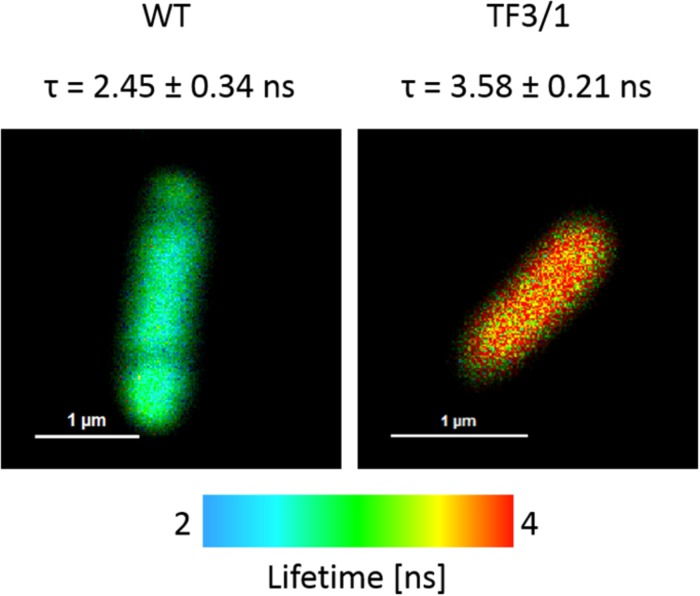
FLIM images of *L. pneumophila* Corby strain and TF3/1 strain cells labeled with SYTO9. The amplitude of averaged fluorescence lifetime values of entire cells is reported (an arithmetic mean from 6 cells ± SD).

**FIGURE 7:**
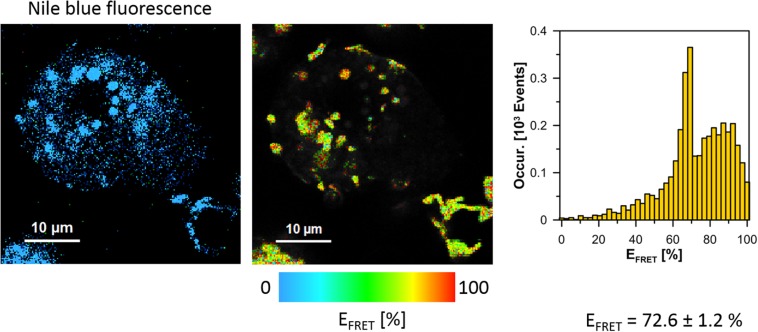
Images illustrating Förster type excitation energy transfer (FRET) between SYTO9 immobilized on the surface of *L. pneumophila* cells (TF3/1 strain) and Nile Blue situated within *A. castellanii* cells. The **left panel** shows a fluorescence image of *A. castellanii* based on selective emission of Nile blue. The **middle panel** shows a map of FRET efficiency (*L. pneumophila* cells appear as bright spots). The **right panel** shows the FRET efficiency histogram representing an analysis of the map shown in the **middle panel**. The average FRET efficiency calculated over histograms in 6 experiments is given ± SD.

**FIGURE 8:**
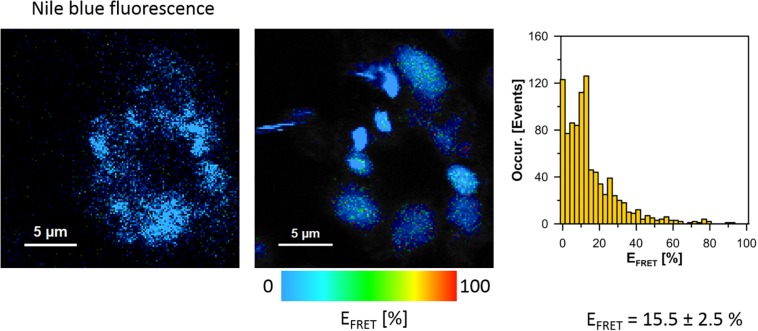
Images illustrating Förster type excitation energy transfer (FRET) between SYTO9 immobilized on the surface of *L. pneumophila* cells (Corby strain) and NB situated within *A. castellanii* cells. The **left panel** shows a fluorescence image of *A. castellanii* based on selective emission of Nile blue. The **middle panel** shows a map of FRET efficiency (*L. pneumophila* cells appear as bright spots). The **right panel** shows the FRET efficiency histogram representing an analysis of the map shown in the **middle panel**. The average FRET efficiency calculated over histograms in 6 experiments is given ± SD.

**FIGURE 9:**
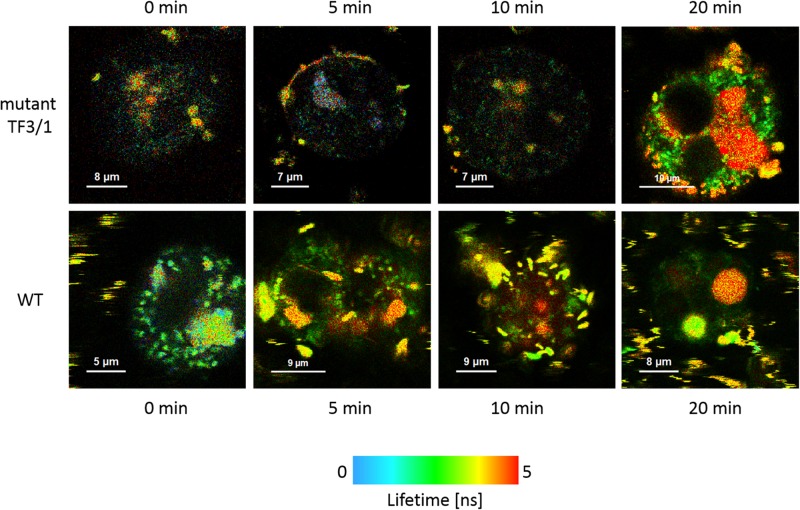
FLIM images of *A. castellanii* cells recorded after different periods (indicated) of exposure to interaction with *L. pneumophila* cells: TF3/1 strain **(upper panel)** and Corby strain **(lower panel)**.

The cells of *L. pneumophila* (Corby strain and TF3/1 strain) were labeled with fluorescence dye SYTO9 and observed in the spectral window 520/35 nm (Supplementary Figure S1). A comparative FLIM analysis of the cells of the Corby strain and the TF3/1 mutant showed substantial differences in the fluorescence lifetimes of the fluorescence probe in both systems (τ = 2.45 ± 0.34 ns vs. 3.58 ± 0.21 ns, respectively, Figure 6). Such a difference points to the LPS-covered cell surface as one of the localizations of SYTO9. Both types of bacterial cells (labeled with SYTO9) were subjected to interaction with *A. castellanii* cells and THP-1 macrophages labeled with Nile blue (NB) and observed in the optical range 690/70 nm (Figures 7, 8 and Supplementary Figure S2). The Förster type excitation energy transfer (FRET) between SYTO9 and NB was applied to monitor the interaction between *L. pneumophila* (TF3/1 strain and Corby strain) and amoeba cells or THP-1 macrophages (Figures 7, 8 and Supplementary Figure S2). In general, limited contact of the Corby strain both with amoeba cells and THP-1 macrophages was observed, as concluded on the basis of the relatively low energy transfer efficiency in fluorescence labels and the low number of occurrence (Figures 8 and Supplementary Figure S2). In the extreme case, no energy transfer has been observed in the THP-1 macrophages interacting with the Corby strain (Supplementary Figure S2). Importantly, the FRET efficiency averaged over the entire histograms was substantially higher in the case of the TF3/1 strain. In the case of the interaction of the *L. pneumophila* TF3/1 strain with amoeba, *E*_*T*_ FRET = 72.6 ± 1.2% vs. *E*_*T*_ FRET = 15.5 ± 2.5% determined for the Corby strain (Figures 7, 8). In the case of the interaction of the *L. pneumophila* TF3/1 strain with THP-1 macrophages, *E*_*T*_ FRET = 14 ± 2% vs. no energy transfer observed for the Corby strain (Supplementary Figure S2). Such a difference can be anticipated taking into consideration the very strong dependency of *E*_*T*_ FRET on the distance (∼R^–6^) and the fact that the polysaccharide chain in the LPS fraction of the TF3/1 mutant is shorter than in the LPS fraction of the *L. pneumophila* Corby strain. The donor-acceptor pair separation was calculated based on spectral properties of both dyes used to label the bacteria and amoeba cells or THP-1 macrophages. Such a distance was determined at *R* = 33.6 Å in the case of the TF3/1 mutant and *R* = 52.4 Å for the Corby strain (Figures 7, 8). Interestingly, the kinetic study of the bacterial interaction with the amoeba (Figure 9) revealed a pronounced difference, i.e., almost instantaneous and highly efficient binding of the wild type cells to the amoeba surface, followed by penetration into the amoeba cells. As shown in Figure 9, such a process was clearly not as efficient in the case of the TF3/1 mutant. The relative amplitude of the fluorescence lifetime of a compound specific for *L. pneumophila* bacteria provides information of the number of bacteria attached to the amoeba surface. This result points to LPS and, in particular, to the length of the polysaccharide fraction as an important *L. pneumophila* determinant involved in the process of adhesion to the host cell.

## Discussion

Attachment of bacteria to eukaryotic cells is a very early and simultaneously crucial step in host–microbe interactions, which determines the further course of infection. Adhesion is a two-step process whose initial phase involves non-specific factors associated with the physicochemical properties of bacteria, e.g., electrostatic potential, hydrophobicity, and van der Wls forces. The key role in adhesion is played by specific interactions with host receptors mediated by bacterial surface structures responsible for the high specificity of the process. Several *L. pneumophila* determinants, with those associated with the cell wall, are known to contribute to adherence to host cells. These include type IV pili, the structural toxin RtxA, the adenylate cyclase LadC, the intergrin analog LaiA, the GAG binding protein Lcl, PilY1 protein, and collagen-like protein (Lcl) (Garduño et al., 1998; Stone and Abu Kwaik, 1998; Cirillo et al., 2001; Chang et al., 2005; Newton et al., 2008; Duncan et al., 2011; Hoppe et al., 2017; Abdel-Nour et al., 2019). Similarly, the *L. pneumophila* major outer membrane protein (MOMP), OmpS protein, and Hsp60 are involved in the attachment to host cells (Garduño et al., 1998, 2002; Krinos et al., 1999).

The architecture of *L. pneumophila* LPS, which is unusual among gram-negative bacteria, results from its specific role in the pathogenicity of these bacteria. The LPS pattern of *L. pneumophila* varies depending on whether the bacteria grew on artificial medium or intracellularly in *Acanthamoeba polyphaga* (Barker et al., 1993). Studies with random mutagenesis showed both flexibility and rigidity of the LPS structure in *L. pneumophila* (Wagner et al., 2007). These bacteria are able to synthesize two functionally different LPS architectures, namely more hydrophobic LPS for survival in aerosols and more hydrophilic LPS for close-packing of legionellae inside clusters (Reichardt et al., 2010). The LPS of *L. pneumophila* is highly hydrophobic due to the presence of deoxy groups and *N*- and *O*-acyl substituents in polylegionaminic acid (Knirel et al., 1994). The highest degree of O-chain hydrophobicity among *L. pneumophila* serotypes is exhibited by strains containing 8-*O*-acetyl groups, which constitute an epitope recognized by Mab3/1 antibodies (Helbig et al., 1995). The presence of 8-*O*-acetyl residues in the O-specific chain in the *L. pneumophila* Corby strain and the trace amounts in the case of the mutant were confirmed using HR-MAS NMR (Figure 3). The trace amounts of high-molecule LPS visible on the gel from the mutant correlate with the NMR data (Figure 2). The presence of two low- and high-molecular weight fractions of LPS explains the presence of two signals of CH_3_ from the N-acetimidoyl group. This indicates that another enzyme, independent of the enzyme encoded by the *lag-1* gene, may play the role of 8-*O* acetyltransferase (Kooistra et al., 2001). The high hydrophobicity of the *L. pneumophila* surface layer facilitates adhesion to the host cell membrane and increases their survival in aerosols. “Hydrophobic bacteria” concentrate preferentially at the interface between the water and air phases of a bubble, which can burst to produce contaminated aerosols. This is regarded as the major mode of the spread of Legionnaires’ disease (Zähringer et al., 1995).

Disturbances in LPS synthesis exert an effect on the synthesis of other cell macromolecules. The *L. pneumophila* TF3/1 mutant type failed to produce high-molecular-weight O-polysaccharide and showed changes in the structure of phospholipids and proteins, compared to the wild type. The differences were mainly noted for the PC and PG classes (Table 2). In the PC class, the mutant type produced PC with molecular weight of 810u and with fatty acid distribution of 20:0/16:1. In turn, in the PG class, the wild type produced PG with molecular weight of 753u and a fatty acid distribution of 20:1/14:1, 16:1/18:1, 17:0cyc/17:0cyc, and 17:1/17:1. TheTF3/1 mutant produced PG with molecular mass 719.83u and with fatty acid distribution of 16:1/16:0 as well as PG with molecular mass 763.76u and with fatty acid distribution of 20:0/15:0, 19:0/16:0. The TF3/1 strain synthesized by 11 mol% lower amounts of branched fatty acids and approximately twofold higher amounts of long-chain fatty acid (20:0) than the Corby strain (Table 1). The differences in the PL and FA between the Corby strain and the TF3/1 strain were confirmed by FTIR spectroscopy. This method also indicated differences between proteins. The Corby strain contained greater abundance of protein β structures (Figure 1).

All these changes in the structure of LPS and other surface components resulted in changes in the adhesive properties of the bacteria. The adhesion assay revealed that the binding of the TF3/1 mutant to *A. castellanii* cells and THP-1 macrophages was reduced by 15% and 18%, respectively, compared to the *L. pneumophila* Corby strain. Similarly, the AFM analyses showed that the mutant exhibited lower adhesion than the wild type; the adhesion was not the same over the whole cell surface and there were areas with greater or lower adhesion. Previous studies have shown that mutations in the *laiA* and *rtxA* genes lead to a 70 and 50% reduction in adhesion to host cells, respectively (Cirillo et al., 2001; Chang et al., 2005).

Attraction and repulsion between polylegionaminic acid and other cell-surface components may stabilize the conformation of macromolecules and the outer membrane as a whole. They can also influence the formation and release of outer membrane vesicles (OMVs). *L. pneumophila* OMVs retain the asymmetric composition of the bacterial outer membrane, i.e., LPS molecules in the outer leaflet and phospholipids in the inner leaflet, and contain a disproportionately high number of virulence-associated proteins (Galka et al., 2008; Jäger et al., 2015). The nano-sized *L. pneumophila* microvesicles play an important role in the interactions with host cells and in the pathogenesis and dissemination of the infection in the organism (Jäger et al., 2014). *L. pneumophila* utilize OMVs to introduce effector molecules into phagosomes to inhibit phagolysosome fusion and formation of an intracellular replication niche (Fernandez-Moreira et al., 2006; Helbig et al., 2006; Galka et al., 2008).

In the present study, the *L. pneumophila* Corby strain produced numerous vesicles with a diameter of ca. 45 nm ± 7 nm on the cell surface, as shown by AFM, which are smaller than the vesicles in the *L. pneumophila* Lp02 strain (Fernandez-Moreira et al., 2006; Figure 5). The TF3/1 strain produced a lower number of vesicles and exhibited grooves on the cell surface. These results may indicate that the LPS structure, in particular the length of the O-specific chain and the structure of PLs with acyl-specific fatty acid residues, may influence the formation of OMVs. Seeger et al. have shown that *L. pneumophila* LPS shed from OMVs is a factor that contributes to the inhibition of phagosome maturation independently from host cell modulating proteins, although there were no differences in the inhibition activity of LPS between the Corby strain and its mutant TF 3/1 (Seeger et al., 2010).

Förster resonance energy transfer was applied to elucidate the role of *L. pneumophila* LPS in the cell-cell interaction in the early phases of infection, i.e., prior to the entry of *L. pneumophila* into the *A. castellanii* cells. This highly sensitive method for studying the kinetics of interactions between cells without altering their physicochemical properties showed higher efficiency of binding of the *L. pneumophila* strain with the full-length LPS O-chain to the amoeba surface, in comparison with the mutant, whose LPS is devoid of the high molecular weight fraction of the O-antigen (Figure 9).

The study revealed that the LPS of *L. pneumophila* serogroup 1 is a major determinant of the cell wall, which anchors bacteria in the host cell membrane. The important role of the O-antigen structure in recognition and uptake by host cells has been indicated by investigations of *Escherichia coli* (O’Donoghue et al., 2017). OMVs devoid of the O-antigen require protein receptors for uptake and use of clathrin-mediated endocytosis as a main route of entry. In contrast, OMVs with an intact O-antigen do not rely on protein receptors for entry, which in turn is mediated by raft-dependent pathways (O’Donoghue et al., 2017). In comparison to the Corby strain, the TF3/1 mutant attached more weakly but entered the amoeba cells, which proves the involvement of adhesins other than LPS in the process.

Despite the differences in the adhesive properties between the Corby strain and its TF3/1 mutant, they showed no difference in intracellular multiplication in *Acanthamoeba castellanii*, U937 cells, and alveolar macrophages of guinea pigs (Lück et al., 2001). The work of Zou et al. (1999) also found no difference in multiplication between the Philadelphia-1 strain and their MAb 3/1 negative mutant. Further studies are required to elucidate whether one *L. pneumophila* adhesion molecule can be replaced by another one or whether there is a need for a multitude of co-operative interactive adhesin-ligand events, well-synchronized in time and space, for productive colonization of the host. The O-antigen constituting the outermost structural region of *L. pneumophila* serogroup 1 LPS is probably the first component in contact with the host cell, which anchors bacteria in host membranes.

## Data Availability Statement

All datasets generated for this study are included in the article/Supplementary Material.

## Author Contributions

MP-S conceived the original research plan. MP-S, RL, AS-C, AS-B, and BK designed and performed the experiments. MP-S, RL, WG, and AC analyzed the data. MP-S, RL, WG, AC, AS-C, CL, and MP wrote and revised the manuscript. All authors read and approved the final manuscript.

## Conflict of Interest

The authors declare that the research was conducted in the absence of any commercial or financial relationships that could be construed as a potential conflict of interest.

## Supplementary Material

FIGURE S1 Normalized absorption (dotted line) and emission (continuous line) spectra of Syto9. Excitation marked with a vertical line and observation with dichroic band-pass filter 520/35.

FIGURE S2 Images illustrating Förster type excitation energy transfer (FRET) between Syto9 labeled *L. pneumophila* cells (TF3/1 and Corby strains) and Nile blue dye labeled THP-1 macrophages. The panel on the **left** shows fluorescence images of THP-1 macrophages based on selective emission of Nile blue. The **middle** panel shows a map of FRET efficiency. The **right** panel shows the FRET efficiency histogram representing an analysis of the map shown in the **middle** panel. The average FRET efficiency calculated over histograms in 2 experiments is given ± SD.
