# Supplementary figures and images for: The Role of Legionella pneumophila Serogroup 1 Lipopolysaccharide in Host-Pathogen Interaction
Source: Front Microbiol. 2019 Dec 17;10:2890. doi: 10.3389/fmicb.2019.02890 (PMC6927915; doi:10.3389/fmicb.2019.02890)

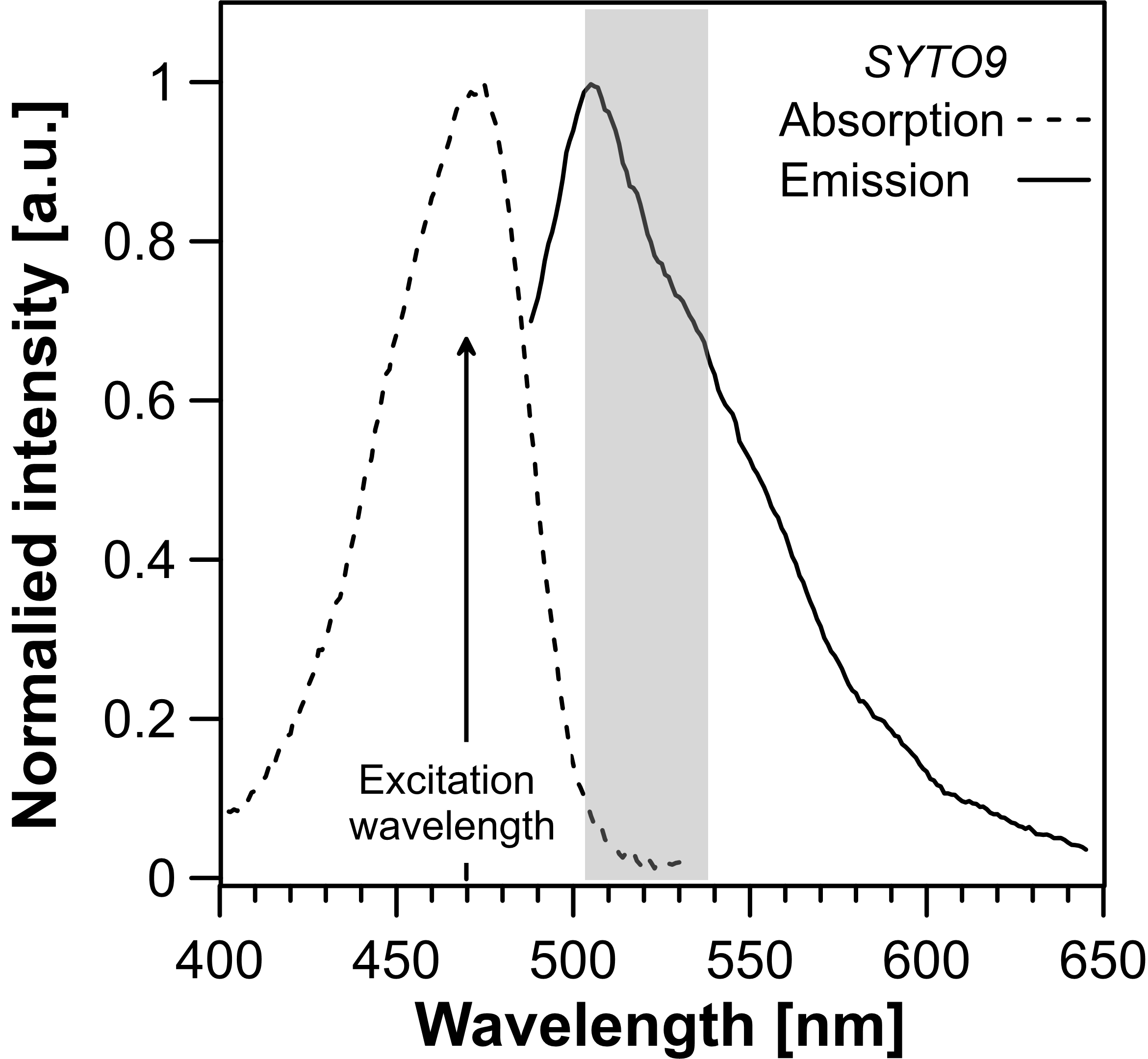

Supplement: FIGURE S1 — Normalized absorption (dotted line) and emission (continuous line) spectra of Syto9. Excitation marked with a vertical line and observation with dichroic band-pass filter 520/35. [file Image_1.TIF]

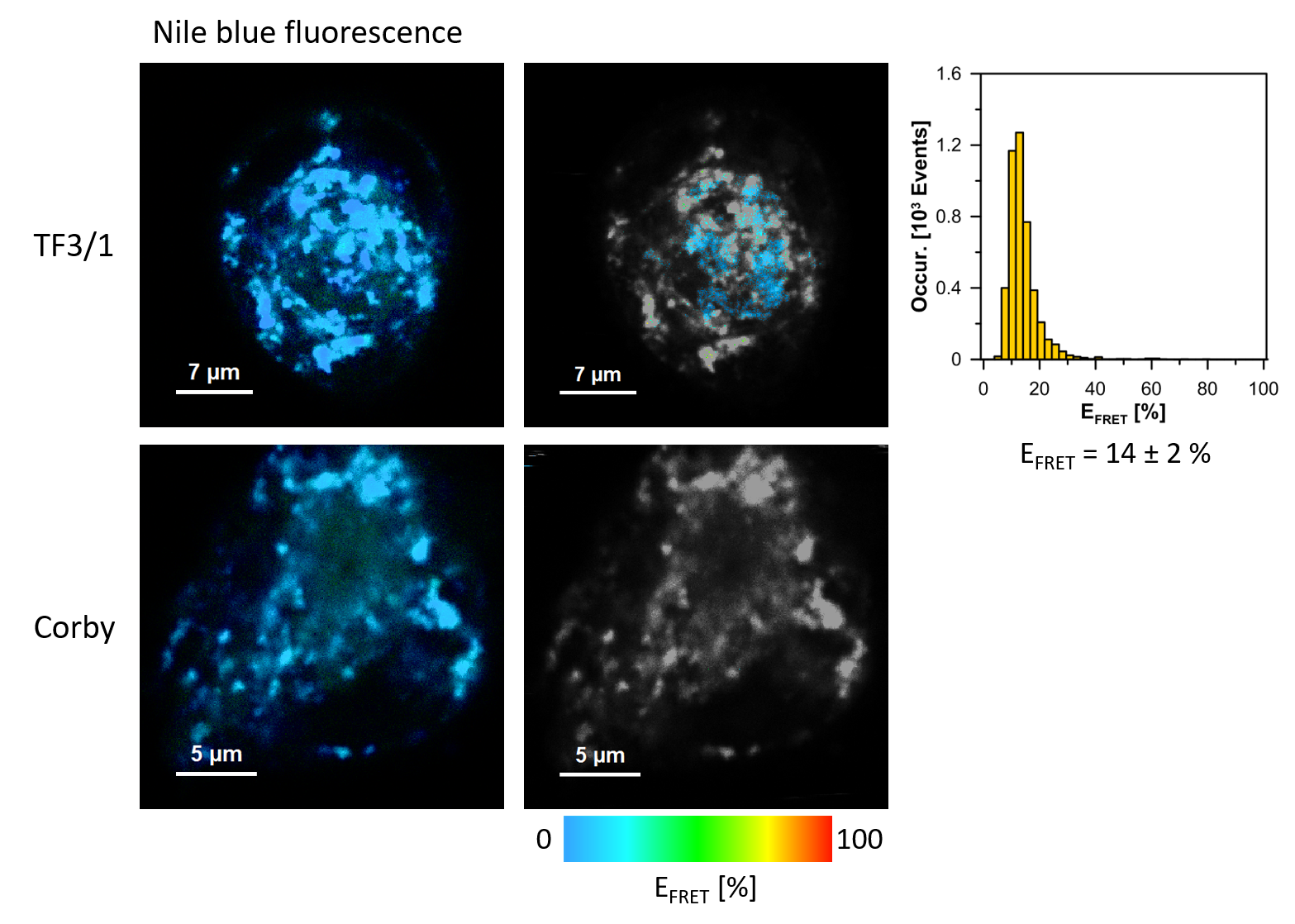

Supplement: FIGURE S2 — Images illustrating Förster type excitation energy transfer (FRET) between Syto9 labeled L. pneumophila cells (TF3/1 and Corby strains) and Nile blue dye labeled THP-1 macrophages. The panel on the left shows fluorescence images of THP-1 macrophages based on selective emission of Nile blue. The middle panel shows a map of FRET efficiency. The right panel shows the FRET efficiency histogram representing an analysis of the map shown in the middle panel. The average FRET efficiency calculated over histograms in 2 experiments is given ± SD. [file Image_2.TIF]
